# Linking sleep apnea and arthritis in the National Alzheimer Coordinating Center Cohort: A cross-sectional analysis

**DOI:** 10.1097/MD.0000000000047717

**Published:** 2026-02-20

**Authors:** Bilal Irfan, Subhamoy Pal, Jonathan Reader, Kelly M. Bakulski, Henry Paulson, Bruno Giordani

**Affiliations:** a Michigan Alzheimer’s Disease Research Center, Ann Arbor, MI; b Department of Neurology, University of Michigan Medical School, Ann Arbor, MI; c Department of Epidemiology, University of Michigan School of Public Health, Ann Arbor, MI; d Center for Bioethics, Harvard Medical School, Boston, MA; e Center for Surgery and Public Health, Brigham and Women’s Hospital, Boston, MA; f Michigan Neuroscience Institute, University of Michigan, Ann Arbor, MI.

**Keywords:** Alzheimer disease, arthritis, cognitive status, sleep apnea

## Abstract

Sleep apnea-related intermittent hypoxia and the chronic inflammation of arthritis share oxidative-stress pathways, yet their epidemiologic overlap remains under-described. The prevalence of both conditions increases with age and presents unique challenges for patient management. To quantify the association between clinician-suspected arthritis and self-reported sleep apnea and to explore whether demographic or cognitive factors modify that link. We analyzed 17,013 adults enrolled in the referral-based National Alzheimer Coordinating Center Uniform Data Set, version 3. Complete-case binary logistic regression modeled obstructive sleep apnea (OSA) (yes/no) on arthritis (yes/no) with adjustment for age, sex, race (White vs non-White), years of education, cognitive status (normal, mild cognitive impairment, Alzheimer disease), body mass index, and cardiometabolic comorbidities. A pre-specified interaction term tested whether cognition modified the arthritis–OSA association. Multiple imputation was used to address missing data. Arthritis was associated with 60% higher odds of OSA (adjusted odds ratio = 1.60, 95% confidence interval: 1.46–1.76, *P* < .001). The effect was attenuated in Alzheimer disease. Male sex, atrial fibrillation, stroke, diabetes, and higher body mass index were additional correlates (all *P* < .001); age was not independently significant. Imputation yielded similar estimates. Clinician-suspected arthritis was robustly associated with self-reported OSA even after extensive adjustment, although unmeasured confounding and exposure misclassification cannot be excluded. Both OSA and arthritis were ascertained by self-report or single-clinician designation without polysomnography, actigraphy, imaging, or serology, raising non-differential misclassification potential. The cross-sectional design prevents causal interpretation, and the predominantly White, highly educated volunteer cohort limits generalizability. Prospective, objectively phenotyped studies, ideally with arthritis sub-typing, are needed to verify directionality and clarify mechanisms. We used records from more than 17,000 volunteers at U.S. Alzheimer Disease Research Centers to ask whether people who say they have arthritis are also more likely to report OSA. After controlling for age, sex, education, cognitive status, weight, and common medical conditions, arthritis still raised the odds of OSA by about 60%. Joint pain and poor sleep can feed off 1 another, so recognizing both problems may help doctors treat them earlier. Neither arthritis nor OSA was confirmed with X-rays, lab tests, or sleep studies, we relied on what participants or clinicians reported. Furthermore, the study looked at 1 point in time, so we cannot tell which problem came 1st; and most volunteers were White and highly educated, so the findings may not reflect every community. Future research that tracks patients over time and uses overnight sleep tests and detailed arthritis subtypes will be crucial.

## 1. Introduction

Sleep apnea, which is often characterized by repeated interruptions in breathing during sleep, is a widely prevalent sleep disorder linked to numerous health conditions, including cardiovascular diseases, diabetes, and arthritis.^[1,2]^ It is marked by repetitive episodes of partial or complete upper-airway obstruction during sleep.^[1,3]^ These interruptions can result in recurrent hypoxia and fragmented sleep, which in turn impact the quality of life and overall health of the affected individuals.^[4]^ Obstructive sleep apnea (OSA) in particular has been associated with several comorbidities, including metabolic disorders and neurocognitive impairments, and is distinctly characterized by its intermittent hypoxia (IH).^[5–7]^ IH among individuals with OSA can trigger oxidative stress and systemic inflammation, which are pivotal in the pathophysiology of the disorder and its associated complications.^[8]^

There is a specific linkage to arthritis, encompassing a range of inflammatory and degenerative joint diseases such as osteoarthritis (OA) and rheumatoid arthritis, which often coexists with sleep disturbances, potentially exacerbating the symptoms of both conditions.^[9–14]^ Arthritis is characterized by chronic joint pain, stiffness, and functional impairment, often leading to decreased mobility and overall negative impacts on the musculoskeletal system.^[15–20]^ Inflammation plays a central role in the pathogenesis of arthritis, with pro-inflammatory cytokines and oxidative stress contributing to joint degradation and pain.^[21–23]^

The intersection between sleep apnea and arthritis has garnered increasing attention in recent years.^[24–26]^ Both conditions share common pathophysiological pathways, including systemic inflammation and oxidative stress.^[27–29]^ The hypoxia–reoxygenation cycles inherent to OSA generate reactive oxygen species (ROS), which, in turn, activate inflammatory pathways.^[30]^ This systemic inflammatory response exacerbates the symptoms of arthritis, leading to a vicious cycle of pain and sleep disruption.^[31–34]^ Studies have demonstrated that individuals with OSA have elevated levels of inflammatory markers such as C-reactive protein, interleukin-6, and tumor necrosis factor-alpha.^[35–37]^ These cytokines are also implicated in the inflammatory processes of arthritis, suggesting a potential bidirectional relationship where each condition potentially aggravates the other.^[38–41]^ For instance, the inflammation and pain associated with arthritis can lead to sleep disturbances, which may predispose individuals to the development of OSA.^[42,43]^ Conversely, the fragmented sleep and IH characteristic of OSA can exacerbate inflammatory processes, worsening arthritis symptoms.^[44–48]^ While the inflammatory mechanism may be shared, the magnitude of risk may differ across arthritis subtypes; however, the dataset we worked with captures only a small number of clinician-flag for arthritis other than OA, necessitating an initial aggregate approach.

The prevalence of both OSA and arthritis increases with age, and they are often found concurrently in older adults.^[49–53]^ The comorbidity of these conditions poses a challenge for patient management and necessitates a multidisciplinary approach to treatment. Understanding the relationship between sleep apnea and arthritis is a crucial step in developing effective therapeutic strategies that serve to address both conditions simultaneously. This study investigates the relationship between sleep apnea and arthritis, examining how demographic and clinical characteristics impact this association using a large cohort from the National Alzheimer Coordinating Center (NACC) Uniform Data Set (UDS). Accordingly, we hypothesized that adults with clinician-assigned arthritis would demonstrate higher odds of self-reported sleep apnea after adjustment for key demographic and cardiometabolic covariates. We also posited that cognitive status (normal, mild cognitive impairment [MCI], Alzheimer disease [AD] ) might modify this association, with stronger effects expected in cognitively normal participants owing to more reliable symptom recognition and reporting.

## 2. Materials and methods

### 2.1. Participants

Participants were recruited from 35 Alzheimer Disease Research Centers (ADRCs), and are enrolled using diverse strategies, such as referrals from clinicians, self-referrals by participants or family members, and community outreach.^[54]^ Many ADRCs also include cognitively normal volunteers, who tend to be highly educated. As a result, the NACC cohort is best considered a referral-based or volunteer case series rather than a population-based sample.^[55]^ All participants provided written informed consent at the time of enrollment and consented to further data analyses. This analysis of existing data was approved by the University of Michigan Institutional Review Board (HUM00000382).

This study used data obtained from the NACC as of August 22, 2023 and consists of data collected between 2005 and 2023, encompassing 17,013 participants from initial assessments with the third version of the UDS version 3 (UDSv3).^[56]^ The cohort included individuals with varying cognitive statuses, including AD, MCI, and normal cognition.^[57]^ Data were collected through standardized assessments covering demographic information, clinical history, cognitive evaluations, and medical and neurological examinations.^[58]^ Preliminary data analysis was presented at the 2024 Alzheimer Association International Conference.^[26]^

### 2.2. Sleep apnea measures

At the initial clinical evaluation, sleep apnea was recorded based on participants’ history of self-reported and/or clinically assessed status (coded as present or absent). These questions were introduced in UDSv3 (implemented in March 2015), which explains the reliance on version 3 data and the significant amount of missing sleep-apnea values.^[59]^ Because this sleep-related information only relatively recently became part of the NACC UDS, there are no objective sleep metrics (e.g., polysomnography [PSG] or actigraphy) available to confirm or refine self-reported sleep apnea.

### 2.3. Arthritis measures

In the NACC UDS, arthritis status is determined by the clinician and recorded using standardized questions. Clinicians specify whether a participant has arthritis (absent/present/unknown), and if present, indicate the arthritis type (e.g., rheumatoid, OA, or other) as well as the anatomical regions affected (e.g., upper extremity, lower extremity, spine).^[60]^ This approach provides a structured, clinician-assigned designation of arthritis without requiring separate laboratory or imaging verification, but allows differentiation by arthritis subtype and bodily location.

### 2.4. Covariate measures

Participants self-reported demographic characteristics including age (in years), race (collapsed in our analysis into White vs non-White), Hispanic origin (yes/no), sex (male, female), educational attainment (in years). Smoking history (yes/no) and alcohol consumption (yes/no) were also self-reported. Cognitive diagnosis (normal cognition, MCI, or AD) was determined through clinical consensus or a single-clinician’s evaluation. Participants self-reported history (yes/no) of the comorbid conditions atrial fibrillation (AF), stroke, and diabetes.^[61]^

### 2.5. Statistical analysis

All analyses were performed using R version 4.5.1 (R Core Team 2024). Participants were included if they had valid, non-missing UDSv3 data for both sleep apnea and arthritis, as well as all covariates (age, race, sex, education, cognitive diagnosis) of interest. Individuals with incomplete or contradictory data on any of these measures were excluded. We assessed for statistical differences of covariates in the whole sample with a 2 sample proportion test (Table 1). We visualized participant inclusion using a flow chart. We described the distributions of participant characteristics in the analytic sample using mean and standard deviation for continuous measures with number and frequency for categorical measures. We provided similar distributions comparing samples by sleep apnea status using chi-square testing for categorical variables and Kruskal–Wallis rank sum test for continuous variables. We restricted the analytic set to participants with complete data on all model variables (“complete-case analysis”), although to determine if the ≤10% missing covariate data impacted findings, a sensitivity analysis was conducted on an imputed dataset.

**Table 1:** Overall demographic and clinical characteristics of the study population.

Characteristic	N = 17,013	*P*-value
Age (mean, SD)	69.1 (10.1)	
Race (%)	Non-White: 22%, White: 78%	**<.001**
Hispanic origin (%)	No: 91%, yes: 9%	**<.001**
Sex (%)	Male: 42.5%, female: 57.5%	**.048**
Education (mean, SD)	16 (2.9)	
Cognitive diagnosis (%)	AD: 25.9%, MCI: 26.1%, normal cognition: 48%	**AD vs normal: .002** **MCI vs normal: .002** AD vs MCI: 1
Body mass index	27.5 (5.6) (missing: 1915)	
Sleep apnea (%)	No: 80.8%, yes: 19.2%	**<.001**
Arthritis (%)	No: 52.6%, yes: 47.4% (missing:121)	.55
Atrial fibrillation (%)	Absent: 94%, present: 6% (missing: 105)	**<.001**
Stroke (%)	Absent: 97.2%, present: 2.8% (missing: 84)	**<.001**
Diabetes (%)	Absent: 86.2%, present: 13.8% (missing: 55	**<.001**
Smoking history (%)	No: 63.2%, yes: 36.8% (missing: 290)	**<.001**
Alcohol consumption (%)	No: 35.5%, yes: 64.5% (missing: 206)	**<.001**

Two sample proportional test.

Bolded *P*-value indicate statistical significance.

AD = Alzheimer disease, MCI = mild cognitive impairment, SD = standard deviation.

Binary logistic regression analysis was employed to examine the association between sleep apnea and arthritis, adjusting for age, race, sex, education, and cognitive status. Additional analyses were conducted to explore the association of other comorbid conditions with the likelihood of sleep apnea. An a priori multiplicative interaction term between arthritis (present/absent) and cognitive status (normal, MCI, AD) was included to test whether the arthritis–OSA association differed by cognition. The results are presented as odds ratios (ORs) with 95% confidence intervals (CIs).

### 2.6. Handling of missing data

We did not impute either the outcome or the independent variable of interest (sleep-apnea status or arthritis status). Continuous covariate body mass index (10% missing) was imputed with predictive mean matching using the Multivariate Imputation by Chained Equations (R package) (mice) package (version 3.17 R). Categorical covariates with <2% missingness (AF, stroke, diabetes, smoking, alcohol) were imputed by mode imputations. A complete-case analysis served as the primary model and the imputed data set as a sensitivity check. Model adequacy was assessed with the Hosmer–Lemeshow goodness-of-fit test (χ^2^ = 12.78, degrees of freedom = 8, *P* = .12) indicating sufficient model fit for the logistic regression.

## 3. Results

The demographic and clinical characteristics of the study population revealed that the mean age of the participants was 69.1 years, with a standard deviation of 10.056 years. The cohort was predominantly White (78%), with 22% identifying as non-White, including 14.9% who identified as Black/African American. However, our analysis was conducted based on a White versus non-White classification. Hispanic origin was reported by 9% of participants. The sex distribution showed a higher proportion of females (57.5%) compared to males (42.5%). The average educational attainment was 16 years (SD = 2.9).

Sleep apnea was present in 19.2% of the participants, while arthritis was reported by 47.4% (Table 1). Cognitive diagnosis within the cohort was diverse, with 25.9% diagnosed with AD, 26.1% with MCI, and 48% having normal cognition (Table 2). The presence of comorbid conditions such as AF, stroke, and diabetes was also recorded, with 6% having AF, 2.8% having a history of stroke, and 13.8% diagnosed with diabetes. 58.6% of those with sleep apnea also had some form of arthritis (Table 3).

**Table 2:** Demographics and clinical characteristics by cognitive health status.

Characteristic	Normal cognition (N = 8174)	MCI (N = 4435)	AD (N = 4404)	*P*-value
Age (mean, SD)	67.5 (10.5)	71.2 (8.7)	69.9 (9.9)	**<.001**
Non-White (%)	26.4%	23.8%	12.1%	**<.001**
Hispanic origin (%)	9.2%	10.8%	6.7%	**<.001**
Male (%)	34.5%	50%	50%	**<.001**
Female (%)	65.5%	50%	50%	**<.001**
Education (mean, SD)	16.3 (2.6)	15.8 (3.0)	15.5 (3.1)	**<.001**
Sleep apnea (%)	17.1%	23%	19.3%	**<.001**
Arthritis (%)	52.1%	50.1%	35.7%	**<.001**
Missing	33	42	46	
Atrial fibrillation (%)	5%	7.4%	6.5%	**<.001**
Missing	35	30	40	
Stroke (%)	1.6%	3.6%	4.2%	**<.001**
Missing	14	17	53	
Diabetes (%)	12.9%	16.6%	12.7%	**<.001**
Missing	20	15	20	
Smoking history (%)	36.5%	39.6%	34.5%	**<.001**
Missing	76	81	133	
Alcohol consumption (%)	70.5%	63.5%	54.3%	**<.001**
Missing	74	49	83	

*Note*: Kruskal–Wallis rank sum test; Pearson Chi-squared test.

Bolded *P*-values indicate statistical significance.

AD = Alzheimer disease; MCI = mild cognitive impairment; SD = standard deviation.

**Table 3:** Demographic and clinical characteristics by sleep apnea status.

Characteristic	No (N = 11,797)	Yes (N = 2818)	*P*-value
Body mass index (mean, SD)	26.8 (5.3)	30.2 (6.2)	**<.001**
Arthritis (%)	46.7%	58.6%	**<.001**
Age (mean, SD)	69.1 (10.1)	69.7 (8.3)	.07
Non-White (%)	22.2%	20.9%	.1
Sex			**<.001**
Male (%)	38.1%	60.2%	
Female (%)	61.9%	39.8%	
Education (mean, SD)	15.9 (2.9)	16.1 (2.7)	**.006**
Cognitive diagnosis (%)	AD: 23.9% MCI: 24.8% Normal cognition:51.3%	AD: 24.6%, MCI: 31.2%, Normal cognition: 44.2%	**<.001**
Atrial fibrillation (%)	5.1%	10.1%	**<.001**
Stroke (%)	2.5%	4.1%	**<.001**
Diabetes (%)	12%	21.7%	**<.001**
Smoking history (%)	36.5%	40.7%	**<.001**
Alcohol consumption (%)	66.2%	63.9%	**.020**

*Note*: Kruskal–Wallis rank sum test; Pearson Chi-squared test.

Bolded *P*-values indicate statistical significance.

AD = Alzheimer disease; MCI = mild cognitive impairment; SD = standard deviation.

The logistic regression model aimed to predict the presence of sleep apnea based on arthritis status, age, race, Hispanic origin, sex, education, and cognitive diagnosis (Table 4). The analysis revealed a significant association between arthritis and sleep apnea, with individuals with arthritis showing 78% higher odds of having sleep apnea (adjusted OR [aOR] = 1.78, 95% CI: 1.55–2.04, *P* < .001).

**Table 4:** Logistic regression model predicting sleep apnea.

Characteristic	aOR	95% CI	*P*-value
Arthritis (yes vs no)	1.78	1.55–2.04	**<.001**
Age	1.00	1.00–1.01	.6
Race (non-White vs White)	0.81	0.72–0.91	**<.001**
Sex (male vs female)	2.42	2.20–2.65	**<.001**
Education	1.05	1.04–1.07	**<.001**
Cognitive diagnosis (AD vs normal)	1.49	1.27–1.75	**<.001**
Cognitive diagnosis (MCI vs normal)	1.49	1.27–1.76	**<.001**
Arthritis* MCI vs arthritis* normal cognition	0.86	0.70–1.07	.2
Arthritis* AD vs arthritis* normal cognition	0.78	0.62–0.97	**.026**
Atrial fibrillation (present vs absent)	1.65	1.40–1.94	**<.001**
Stroke (present vs absent)	1.50	1.18–1.90	**<.001**
Diabetes (present vs absent)	1.52	1.35–1.71	**<.001**
Smoking history (yes vs no)	1.07	0.98–1.17	.2
Alcohol consumption (yes vs no)	0.93	0.84–1.02	.1
Body mass index	1.11	1.10–1.12	**<.001**

*Note*: Bolded *P*-values indicate statistical significance.

AD = Alzheimer disease, aOR = adjusted odds ratio, CI = confidence interval, MCI = mild cognitive impairment, SD = standard deviation.

Age did not show a significant association with sleep apnea (aOR = 1.00, 95% CI: 1.00–1.01, *P* = .6), suggesting that while sleep apnea prevalence increases with age, other factors might be more influential in this cohort. Non-White individuals had lower odds of sleep apnea compared to White individuals (aOR = 0.81, 95% CI: 0.72–0.91, *P* = <.001). Sex differences were pronounced, with males significantly more likely to have sleep apnea compared to females (aOR = 2.42, 95% CI: 2.20–2.65, *P* < .001). Educational attainment showed a significant positive correlation with the presence of sleep apnea (aOR = 1.05, 95% CI: 1.04–1.07, *P* = <.001).

Sensitivity analyses using the imputed data set yielded virtually identical effect estimates (e.g., arthritis aOR = 1.77, 95% CI: 1.56–2.01, *P* = <.001 in the imputed model compared to arthritis aOR = 1.78, 95% CI: 1.55–2.04, *P* < .001) and significance patterns (Table 5). Thus, missing-data imputation did not alter our substantive conclusions and thus we report results from the complete-case analysis.

**Table 5:** Logistic regression model predicting sleep apnea after imputation of missing values.

Characteristic	aOR	95% CI	*P*-value
Arthritis (yes vs no)	1.77	1.56–2.01	**<.001**
Age	1.00	1.00–1.01	.5
Race (non-White vs White)	0.83	0.75–0.92	**<.001**
Sex (male vs female)	2.38	2.19–2.59	**<.001**
Education	1.05	1.03–1.06	**<.001**
Cognitive diagnosis (AD vs normal)	1.44	1.24–1.66	**<.001**
Cognitive diagnosis (MCI vs normal)	1.46	1.26–1.70	**<.001**
Arthritis* MCI vs arthritis*normal cognition	0.89	0.73–1.08	.2
Arthritis* AD vs arthritis*normal cognition	0.76	0.62–0.93	**.007**
Atrial fibrillation (present vs absent)	1.59	1.36–1.84	**<.001**
Stroke (present vs absent)	1.38	1.10–1.71	**.004**
Diabetes (present vs absent)	1.56	1.40–1.73	**<.001**
Smoking history (yes vs no)	1.04	0.96–1.14	.3
Alcohol consumption (yes vs no)	0.92	0.84–1.00	.056
Body mass index	1.11	1.10–1.12	**<.001**

*Note*: Bolded *P*-values indicate statistical significance.

AD = Alzheimer disease; CI = confidence interval; MCI = mild cognitive impairment; SD = standard deviation.

Diagnosis had a correlation with sleep apnea. Individuals with MCI had higher odds of having sleep apnea compared to those with normal cognition (aOR = 1.49, 95% CI: 1.27–1.76, *P* < .001). Similarly, those with AD also showed increased odds over normal cognition (aOR = 1.49, 95% CI: 1.27–1.75, *P* < .001). The presence of AF was another significant factor, with participants having recent or remote AF showing higher odds of sleep apnea (aOR = 1.65, 95% CI: 1.40–1.94, *P* < .001). Stroke history also contributed to increased sleep apnea odds (aOR = 1.50, 95% CI: 1.18–1.90, *P* < .001).

Diabetes was strongly linked to sleep apnea, with diabetic individuals showing the higher odds of having sleep apnea (aOR = 1.52, 95% CI: 1.35–1.71, *P* < .001). Smoking history showed a non-significant increase in sleep apnea odds (aOR = 1.07, 95% CI: 0.98–1.17, *P* = .15). Alcohol consumption also had no effect on sleep apnea (aOR = 0.93, 95% CI: 0.84–1.02, *P* = .11).

**Figure 1.**
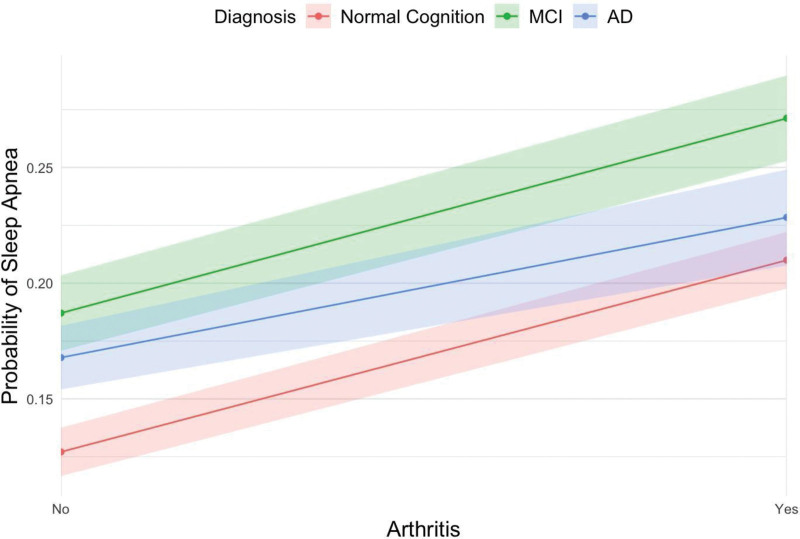
Interaction plot of arthritis and diagnosis.

The interaction between arthritis and cognitive status was also assessed and found to be significant such that although the risk of sleep apnea increases for all individuals who have arthritis, this effect is attenuated for individuals with AD compared to individuals with normal cognition (aOR = 0.78, 95% CI: 0.62–0.97, *P* = .026) (Fig. 1).

Detailed analysis of the study population by cognitive diagnosis revealed further insights. Participants with AD were older on average (mean age 69.9 years) compared to those with normal cognition (mean age 67.5 years) but not to those with participants with MCI (mean age 71.2 years). The prevalence of arthritis varied significantly across cognitive groups, being least prevalent in the AD group (35.7%) and most prevalent in the normal cognition group (52.1%).

## 4. Discussion

The findings of this study underscore a clearly significant association between arthritis and sleep apnea, which calls upon the need for integrated management strategies for patients with both conditions. The association between arthritis and sleep apnea may be driven by shared inflammatory pathways, especially in the case of rheumatoid arthritis and psoriatic arthritis, which warrant further investigation to understand the potential intricate biological interactions at play that may also be impacting the noted sex differences.^[62]^

Several mechanisms underpin the relationship between sleep apnea and arthritis.^[63,64]^ The chronic IH experienced by OSA patients leads to the generation of ROS, which causes oxidative stress that is a critical factor in the pathogenesis of both OSA and arthritis.^[7,65–68]^ ROS can damage cellular components, leading to the activation of nuclear factor-kappa B, a transcription factor that promotes the expression of pro-inflammatory cytokines.^[69–71]^ The resulting systemic inflammation can contribute to the exacerbation of joint pain and degradation in arthritis.^[72,73]^ Additionally, OSA-induced hypoxia can lead to endothelial dysfunction and vascular inflammation, further compounding the systemic inflammatory state.^[74,75]^ Endothelial dysfunction is a precursor to atherosclerosis, which is commonly observed in OSA patients and is also prevalent in individuals with arthritis.^[76–79]^ The shared inflammatory pathways between these conditions highlight the importance of managing inflammation to mitigate the adverse effects of both sleep apnea and arthritis.

Individuals with MCI and AD having higher odds of sleep apnea than do those with normal cognition, which could reflect mechanisms linking cognitive decline and sleep apnea that involve neuroinflammation, changes in brain structure, and/or overall disruptions in sleep architecture as a consequence.^[80–84]^ Some of the observed sex differences may be attributable to the fact that men tend to have larger neck circumferences and greater upper body obesity, both of which are risk factors for OSA.^[85–90]^ This finding aligns with previous studies suggesting that sleep apnea is more prevalent in males, potentially due to anatomical and hormonal differences.^[91]^ Furthermore, the protective effects arising from progesterone in premenopausal women could serve to contribute to their lower prevalence rates.^[92,93]^ Contrary to population-based studies, age was not independently associated with OSA in this volunteer cohort.^[94,95]^ Selective referral of younger individuals with symptomatic OSA and exclusion of very old participants (due to missing sleep items) (the mean age of those with missing is 72 years) may have attenuated the gradient.

One counterintuitive finding in this study is the protective effect of alcohol consumption against sleep apnea, with those consuming alcohol daily or almost daily having lower odds compared to less frequent drinkers. While excessive alcohol use is generally detrimental to sleep quality, this finding may reflect complex interactions between alcohol use and sleep architecture.^[96–101]^ A meta-analysis of 21 studies found that higher rates of alcohol consumption increased the risk of sleep apnea by 25%.^[96]^ It is possible that moderate alcohol consumption before bed might induce sedation and muscle relaxation, temporarily reducing the frequency of apneic events, though significant conflicting evidence exists.^[102]^ This point merits further investigation, as the overall impact of alcohol on sleep apnea is generally considered negative. The positive education–OSA association likely reflects ascertainment bias: participants with higher educational attainment may be more health-literate, more likely to seek sleep evaluation, and thus more likely to self-report a prior diagnosis.

These results emphasize the importance of screening for sleep apnea in patients with arthritis, particularly those with additional risk factors such as AF, stroke, or diabetes.^[103]^ The presence of these conditions likely exacerbates the severity of sleep apnea, as displayed by the presence of its hallmarks, nocturnal hypoxia and fragmented sleep, commonly observed in cases of AF.^[104,105]^ Similarly, diabetes and obesity, common in both sleep apnea and arthritis patients, contribute to systemic inflammation and metabolic dysregulation, further complicating the clinical picture.^[106,107]^ The relationship between diabetes and sleep apnea is well-documented, as both conditions share common risk factors such as obesity and insulin resistance, and they may exacerbate each other’s severity.^[108]^ It is important to note that visceral adiposity can promote insulin resistance and chronic low-grade inflammation through increased secretion of pro-inflammatory adipokines (such as leptin and resistin) and reduced production of anti-inflammatoryadiponectin, which can thereby serve to amplify endothelial dysfunction and oxidative stress. In turn, recurrent hypoxia and sleep fragmentation in OSA can actually further worsen insulin resistance and glucose intolerance, while hyperglycemia and metabolic dysregulation may adversely affect upper-airway neuromuscular control and ventilatory drive. These interrelated mechanisms can help explain, in part, why obesity, diabetes, OSA, and arthritis frequently cluster clinically and may jointly exacerbate pain, limit mobility functions, and increase cardiometabolic risk. Healthcare providers should be aware of the increased risk and consider early intervention strategies to mitigate the adverse effects of sleep apnea on overall health and quality of life, and the need for socioculturally conscious care strategies across diverse population groups.^[109–113]^

### 4.1. Strengths and limitations

The strengths of this study include the large sample size and the comprehensive assessment of demographic and clinical variables, though it is limited by the cross-sectional design which precludes causal inferences.^[114–118]^ Additionally, the presence of missing data for certain variables may introduce bias, although the large sample size helps to mitigate this issue.^[119–121]^ Nevertheless, our multiple-imputation sensitivity analysis confirmed that key OR estimates changed only by 1%, suggesting that missingness did not materially bias results.

Because ADRC volunteers are predominantly White and well-educated, the cohort is not population-representative. Findings should therefore be extrapolated with caution to under-represented racial or ethnic groups, and to individuals with lower educational attainment. Our race dichotomization, while pragmatic due to a small representation of individual minority groups in the sample, may mask heterogeneity (e.g., under-diagnosis of OSA among Black adults); future studies with larger diverse samples should examine subgroup-specific risks. Furthermore, women can also be under-diagnosed for OSA due to presenting different nonspecific symptoms such as insomnia, depressive episodes, fatigue, nightmares, headaches, and changes around the menopausal transition.^[122]^ There are other limitations as well that need to be noted. For example, both sleep apnea and arthritis were ascertained through self-report or single-clinician designation, without objective validation. Although prior work suggests moderate agreement between self-report and PSG for moderate-to-severe OSA, we cannot exclude exposure misclassification.^[123]^ Given the likely non-differential nature of such error, our effect estimates are plausibly under-than over-estimated.

Furthermore, because neither PSG nor actigraphy are available in UDSv3, the possibility of non-differential misclassification of OSA status must be recognized; any such misclassification would bias associations towards the null. Similarly, the absence of imaging or serological confirmation likewise introduces potential misclassification, particularly between OA and inflammatory arthritides. We therefore interpret our results as applying to “clinician-suspected arthritis” rather than to pathologically confirmed disease. Because the UDS captures arthritis only as a clinician-flag denoting OA, rheumatoid, and classifies all other ones such as psoriatic, gouty, or other inflammatory subtypes as “other,” the association we report represents a pooled average across heterogeneous joint diseases, potentially diluting subtype-specific effects and thereby constraining both the generalizability of the findings to distinct arthritis populations and the pathophysiological specificity that future, subtype-resolved studies could achieve.

In addition, several biologically relevant covariates were not available in UDSv3 and therefore could not be modeled. Although overall adiposity was approximated by body mass index, we lacked more discriminating measures of fat distribution (e.g., neck circumference, visceral adiposity) and we could not ascertain menopausal status, exogenous hormone use, or circulating androgen and estrogen levels. These unmeasured factors plausibly influence both upper-airway collapsibility and systemic inflammation, and their omission may have contributed to the larger male-female differential we observed. Residual confounding by obesity phenotypes or endocrine status could thus either exaggerate or attenuate true sex-specific associations, showcasing the need for replication in cohorts with detailed anthropometric and hormonal data.

### 4.2. Implications

While our cross-sectional design precludes causal inference, the observed association and the co-occurrence of sleep apnea and arthritis has significant clinical implications. Patients with both conditions often experience a reduced quality of life due to the synergistic effects of pain, sleep disruption, and inflammation. Because our analyses rely on self-reported exposure and outcome and capture a single cross-sectional time-point, the practice suggestions below should be viewed as hypothesis-generating rather than guideline-setting. Nevertheless, the consistent association of arthritis with greater odds of sleep apnea indicates that clinicians may wish to maintain a low threshold for objective sleep evaluation (e.g., home sleep-apnea testing or PSG) in symptomatic arthritis patients, especially when additional cardiometabolic comorbidity is present, while recognizing that effective sleep-apnea therapies such as continuous positive airway pressure are likely to yield benefits that management of joint disease alone cannot achieve. Effective management requires an approach comprehensive in scope, 1 that addresses both the sleep disorder and the inflammatory condition. Continuous positive airway pressure therapy, the gold standard treatment for OSA, has been shown to reduce inflammation and improve cardiovascular outcomes, potentially alleviating some symptoms of arthritis.^[124–127]^ Conversely, optimizing the management of arthritis through anti-inflammatorymedications and physical therapy may improve sleep quality and reduce the severity of OSA. Recognizing the association that is potentially bidirectional, although causality cannot be inferred from cross-sectional data, between these conditions is essential for developing holistic treatment plans that enhance patient outcomes. The variation in arthritis prevalence across cognitive groups may suggest that arthritis is more frequently diagnosed or reported among cognitively normal individuals or that cognitive decline in conditions like AD may impact the recognition and management of arthritis symptoms.^[128–130]^ Cognitive impairment could affect patients’ ability to communicate pain or seek treatment, leading to under-diagnosis or less frequent management of arthritis in this population.^[131]^

## 5. Conclusions

Future studies would benefit from exploring the potential longitudinal impact of sleep apnea on arthritis progression and any benefits arising from targeted interventions. This can involve an investigation into the underlying mechanisms that link these conditions, as it will be crucial for developing effective treatments in light of the associations found between arthritis and sleep apnea within the course of this study.

## Author contributions

**Conceptualization:** Bilal Irfan, Subhamoy Pal, Jonathan Reader, Bruno Giordani.

**Data curation:** Bilal Irfan, Subhamoy Pal, Jonathan Reader, Bruno Giordani.

**Formal analysis:** Bilal Irfan, Subhamoy Pal, Jonathan Reader, Bruno Giordani.

**Methodology:** Bilal Irfan, Subhamoy Pal, Jonathan Reader, Bruno Giordani.

**Resources:** Bilal Irfan, Subhamoy Pal, Jonathan Reader, Bruno Giordani.

**Software:** Bilal Irfan, Subhamoy Pal, Jonathan Reader, Bruno Giordani.

**Supervision:** Bruno Giordani.

**Writing – original draft:** Bilal Irfan, Subhamoy Pal, Jonathan Reader, Bruno Giordani.

**Writing – review & editing:** Bilal Irfan, Subhamoy Pal, Jonathan Reader, Kelly M. Bakulski, Henry Paulson, Bruno Giordani.
